# Knowledge framework of intravenous immunoglobulin resistance in the field of **Kawasaki** disease: A bibliometric analysis (1997−2023)

**DOI:** 10.1002/iid3.1277

**Published:** 2024-05-22

**Authors:** Jiaying Zhang, Hongbiao Huang, Lei Xu, Shuhui Wang, Yang Gao, Wenyu Zhuo, Yan Wang, Yiming Zheng, Xuan Tang, Jiaqi Jiang, Haitao Lv

**Affiliations:** ^1^ Institute of Pediatric Research Children's Hospital of Soochow University Suzhou Jiangsu China; ^2^ Department of Pediatrics Fujian Province Hospital Fuzhou Fujian China; ^3^ Department of Pediatrics, No.2 Affiliated Hospital Air Force Medical University Xian Shanxi China

**Keywords:** bibliometrics, intravenous immunoglobulin resistance, Kawasaki disease

## Abstract

**Background:**

Kawasaki disease (KD) is an autoimmune disease with cardiovascular disease as its main complication, mainly affecting children under 5 years old. KD treatment has made tremendous progress in recent years, but intravenous immunoglobulin (IVIG) resistance remains a major dilemma. Bibliometric analysis had not been used previously to summarize and analyze publications related to IVIG resistance in KD. This study aimed to provide an overview of the knowledge framework and research hotspots in this field through bibliometrics, and provide references for future basic and clinical research.

**Methods:**

Through bibliometric analysis of relevant literature published on the Web of Science Core Collection (WoSCC) database between 1997 and 2023, we investigated the cooccurrence and collaboration relationships among countries, institutions, journals, and authors and summarized key research topics and hotspots.

**Results:**

Following screening, a total of 364 publications were downloaded, comprising 328 articles and 36 reviews. The number of articles on IVIG resistance increased year on year and the top three most productive countries were China, Japan, and the United States. *Frontiers in Pediatrics* had the most published articles, and the *Journal of Pediatrics* had the most citations. IVIG resistance had been studied by 1889 authors, of whom Kuo Ho Chang had published the most papers.

**Conclusion:**

Research in the field was focused on risk factors, therapy (atorvastatin, tumor necrosis factor‐alpha inhibitors), pathogenesis (gene expression), and similar diseases (multisystem inflammatory syndrome in children, MIS‐C). “Treatment,” “risk factor,” and “prediction” were important keywords, providing a valuable reference for scholars studying this field. We suggest that, in the future, more active international collaborations are carried out to study the pathogenesis of IVIG insensitivity, using high‐throughput sequencing technology. We also recommend that machine learning techniques are applied to explore the predictive variables of IVIG resistance.

## Graphical abstract

(1) We summarized the most productive countries/regions, institutions, journals, and authors in the field of IVIG resistance in Kawasaki disease (KD) by bibliometric methods. (2) We found that research on IVIG resistance in KD mainly focuses on the pathogenesis, treatment, risk factors, and related diseases multisystemic inflammatory syndrome in children (MIS‐C). The emerging research directions are treatment, risk factors, and predictive models. (3) We suggest that more active international collaborations are carried out to study the pathogenesis of IVIG insensitivity using high‐throughput sequencing technology. We also recommend that machine learning techniques are applied to explore the predictive variables of IVIG resistance.

## 1 INTRODUCTION

Kawasaki disease (KD) is an acute febrile illness that usually occurs in young children and primarily damages the cardiovascular system.^1^ It is reported that the disease mainly involves medium‐sized blood vessels, especially the coronary arteries. If not treated promptly, it may develop into arterial dilatation and aneurysms, followed by stenosis, obstruction, and even death.^2^ The incidence of coronary artery injury in KD has decreased from 25% to 4% with the use of intravenous immunoglobulin (IVIG) in the treatment of the disease. However, 10%−20% of people are not sensitive to IVIG, which manifests as recurrent or persistent fever in KD, and these patients are more prone to coronary artery injury.^1^, ^3^ In addition, such patients may develop serious complications, such as KD shock syndrome, which can be life‐threatening in severe cases.^4^ Early identification of IVIG‐resistant patients and the addition of corticosteroids on initial treatment may reduce the number of KD patients presenting with IVIG insensitivity.^5^ In particular, since the global outbreak of the 2019 coronavirus disease (COVID‐19), multisystemic inflammatory syndrome in children (MIS‐C) has been seen in many, which has raised concerns among clinicians and scientists. The symptoms of MIS‐C are highly similar to KD, with some patients meeting fully the diagnostic criteria for KD.^6^ For IVIG‐resistant MIS‐C, experts suggest that corticosteroids, anakinra, and infliximab can be used for treatment.^7^, ^8^, ^9^, ^10^ Despite the increasing number of published articles on IVIG resistance, there has been a lack of effective integration and analysis to reflect the main research topics and future research directions.

Bibliometric analysis is a method of studying publications and analyzing the contributions of countries, institutions, and authors.^10^ Notably, it also plays an important role in predicting future research directions.^11^ At present, VOSviewer,^12^ CiteSpace,^13^ R package “Bibliometrix,”^14^ and other tools are widely used in the field of bibliometrics. Bibliometric analysis has been used in previous studies in many fields, such as cardiovascular,^15^ autoimmune,^16^ and metabolic diseases.^17^ Previously published articles have summarized hot topics and cutting‐edge issues in the field of KD in recent years.^18^, ^19^ However, the literature on IVIG resistance in KD has not been integrated by bibliometrics. Therefore, this study aimed to summarize the existing research results and explore future research trends in the field. The following aspects were analyzed: (1) the most creative countries, institutions, journals, and authors in the field; (2) main research directions and hot topics; and (3) future research trends in IVIG resistance.

## 2 MATERIALS AND METHODS

### 2.1 Study procedure

Data for this study were obtained from the Web of Science Core Collection (WoSCC) database. The search formula used was as follows: TS = (Kawasaki disease OR Kawasaki Syndrome OR mucocutaneous lymph node syndrome) AND TS = (IVIG resistance OR IVIG unresponsiveness). Papers published between January 1, 1997 and July 22, 2023 were included.

### 2.2 Inclusion and exclusion criteria

The article types were set to “article” and “review,” and only articles in English were selected. Papers that contained meeting abstracts, early access articles, letters, papers of conference proceedings, and editorial material were excluded.

### 2.3 Data analysis

**Figure 1:**
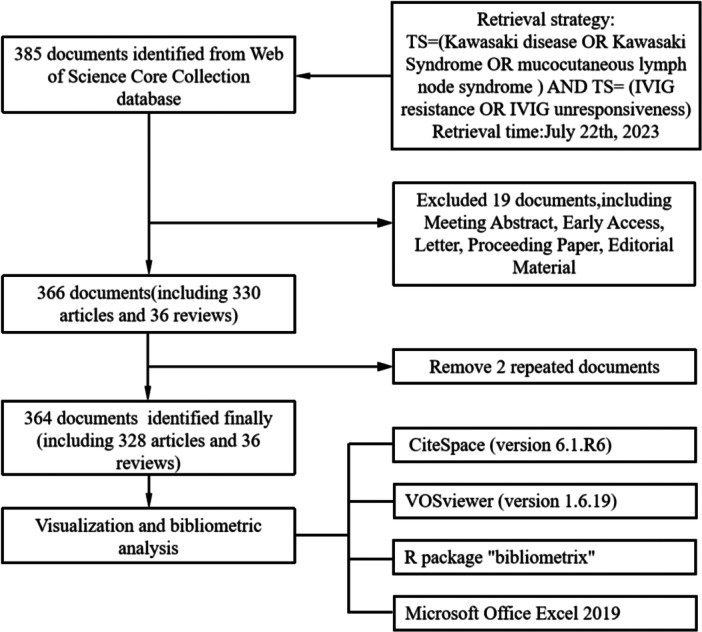
Search strategy and analysis of publications.

We used CiteSpace (version 6.1.R6), VOSviewer (version 1.6.19), the R package Bibliometrix, and Microsoft Office Excel 2019 for bibliometric and visual analysis. We utilized Microsoft Office Excel 2019 for data processing, visual analysis of publications, and citation trends. CiteSpace was used for institutional collaboration, dual‐map overlay of journals, reference analysis, and keyword timeline graph. In addition, VOSviewer was applied to the analysis of regions and countries, institutional analysis, journal, and co‐cited journal analysis, author and co‐cited author analysis, co‐cited reference analysis, and keyword analysis. The cooperation between countries was mapped using Bibliometrix (Figure 1).

## 3 RESULTS

### 3.1 Analysis of publications

**Figure 2:**
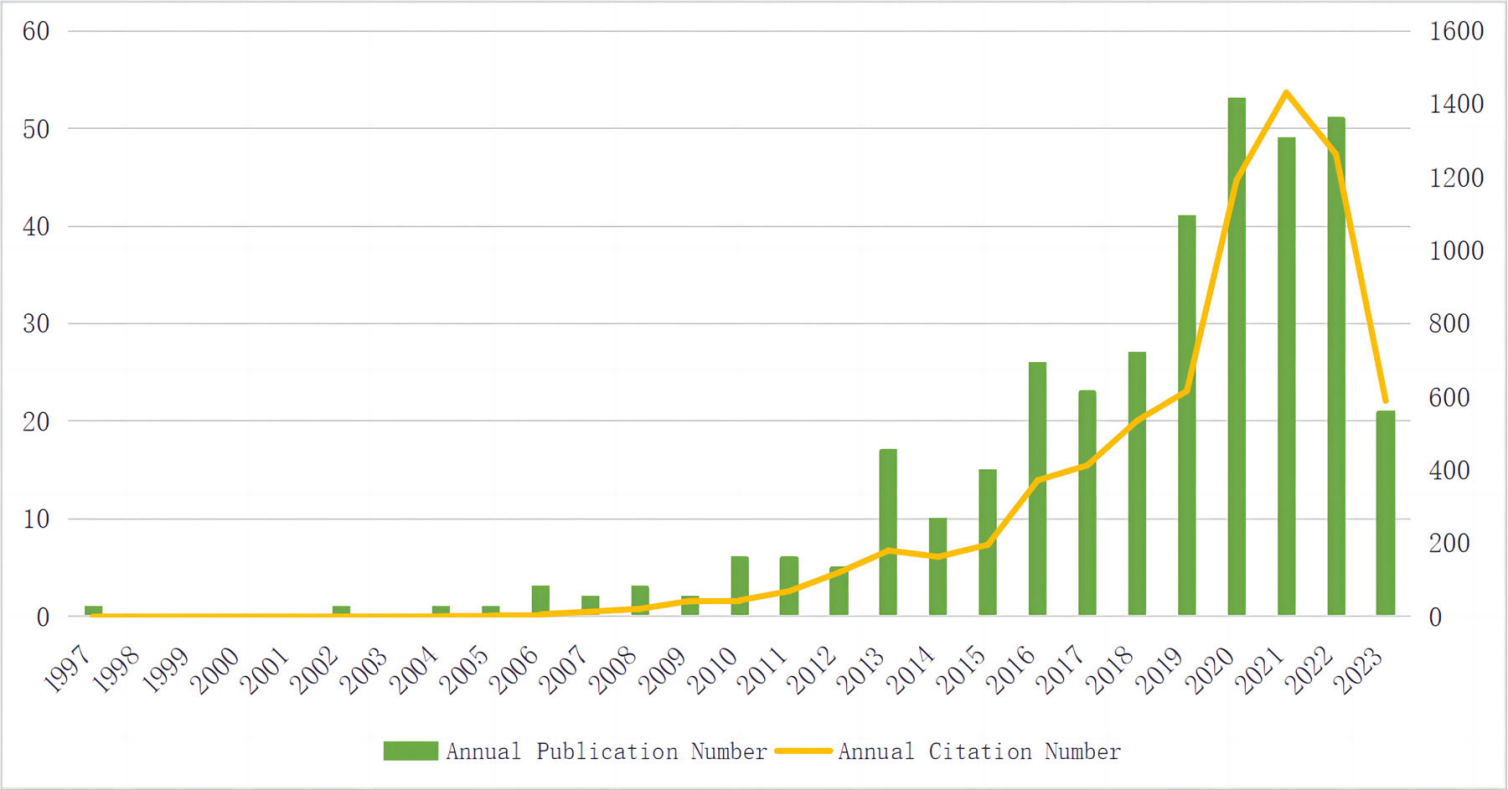
Annual trends in publications and citations.

According to our retrieval strategy, two duplicate papers were excluded, and 364 relevant papers were obtained, including 328 articles and 36 reviews. Overall, the number of publications increased year by year (Figure 2). Based on the number of publications, we categorized the progression into three stages. In the first phase (1997−2009), there were relatively few articles, indicating that research in this field was still nascent. In the second phase (2010−2018), the number of publications increased steadily. In the third phase (2019−2022), the number of articles saw a significant increase. The year 2020 was the most productive, with 53 publications. These publications were cited a total of 7269 times, with an average of 19.97 citations per article. Additionally, the annual number of citations for relevant literature in this field has been on the rise. Notably, more than half of the citations occurred between 2020 and 2022, accounting for 3886 citations or 53.46%. These numbers indicate that scholars are focusing increasingly on the field of IVIG resistance.

### 3.2 Analysis of countries/regions

A total of 42 countries/regions participated in studies of IVIG resistance in KD. Table 1 lists the top 10 countries with the highest output of studies. Among these countries/regions, China published the most articles (*n* = 153), followed by Japan (*n* = 87) and the United States (*n* = 49). The countries with the highest frequency of citations were China (*n* = 2157), Japan (*n* = 2108), and the United States (*n* = 1675). Although China contributed the most to the number of publications, France ranked first in citations per article.

**Table 1:** Top 10 countries/regions in the research field of IVIG resistance in Kawasaki disease.

Rank	Country/region	Publications (*n*)	Citations (*n*)	Average number of article citations
1	China	153	2157	14.10
2	Japan	87	2108	24.23
3	USA	49	1675	34.18
4	South Korea	29	364	12.55
5	Canada	12	399	33.25
6	Italy	12	308	25.67
7	India	7	82	11.71
8	Turkey	6	45	7.50
9	France	5	395	79.00

**Figure 3:**
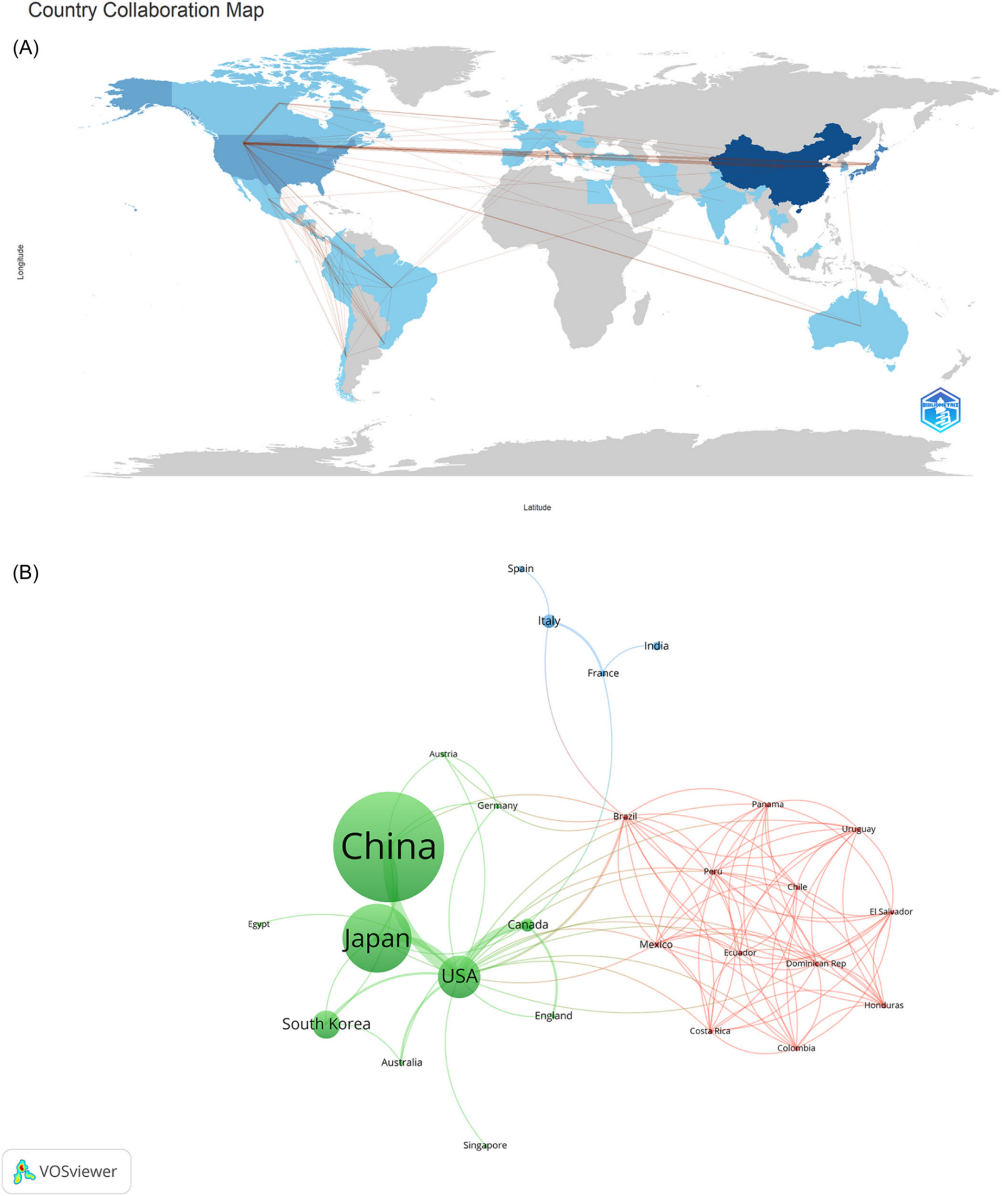
Analysis of countries/regions. (A) Distribution of countries/regions. (B) Analysis of cooperation networks between countries/regions.

Cooperation between the different countries/regions is shown in Figure 3A. As can be seen from the figure, the most active countries were mainly located in North America, South America, and Asia. In VOSviewer, the size of the node correlates with the productivity of the country (Figure 3B). The thickness of the lines represents the intensity of cooperation between countries. China, Japan, and the United States, which published the most articles, belong to the green cluster and occupy the central position. Therefore, these three countries were the main contributors to research on IVIG resistance in KD for the study period. We also found that the United States had close cooperation with Japan, China, Canada, and South Korea in this field of study.

### 3.3 Analysis of institutions

A total of 590 institutions participated in the study of IVIG resistance in KD. The top 10 institutions in terms of number of publications were located in three countries, with six in China, two in the United States, and two in Japan (Table 2). Chang Gung University in China was the most active institution with 32 publications, followed by Chang Gung Memorial Hospital (*n* = 30), University of California, San Diego (*n* = 15), and Sichuan University (*n* = 12). The most frequently cited institutions were Boston Children's Hospital (*n* = 789), the University of California, San Diego (*n* = 707), and Chang Gung Memorial Hospital (*n* = 661). Although China had six institutions on the list, the institution with the highest citation per article was Boston Children's Hospital in the United States.

**Table 2:** Top 10 institutions in the research field of IVIG resistance in Kawasaki disease.

Rank	Institution	Publications (*n*)	Citations (*n*)	Average number of article citations	Total link strength
1	Chang Gung University (China)	32	629	19.66	49
2	Chang Gung Memorial Hospital (China)	30	661	22.03	46
3	University of California San Diego (USA)	15	707	47.13	19
4	Sichuan University (China)	12	86	7.17	2
5	Guangzhou Medical University (China)	11	61	5.55	0
6	Soochow University (China)	11	129	11.73	0
7	Boston Children's Hospital (USA)	10	789	78.90	6
8	University of Tokyo (Japan)	10	40	4.00	22
9	Chong Qing Medical University (China)	9	262	29.11	3
10	National Center for Child Health and Development (Japan)	9	131	14.56	16

**Figure 4:**
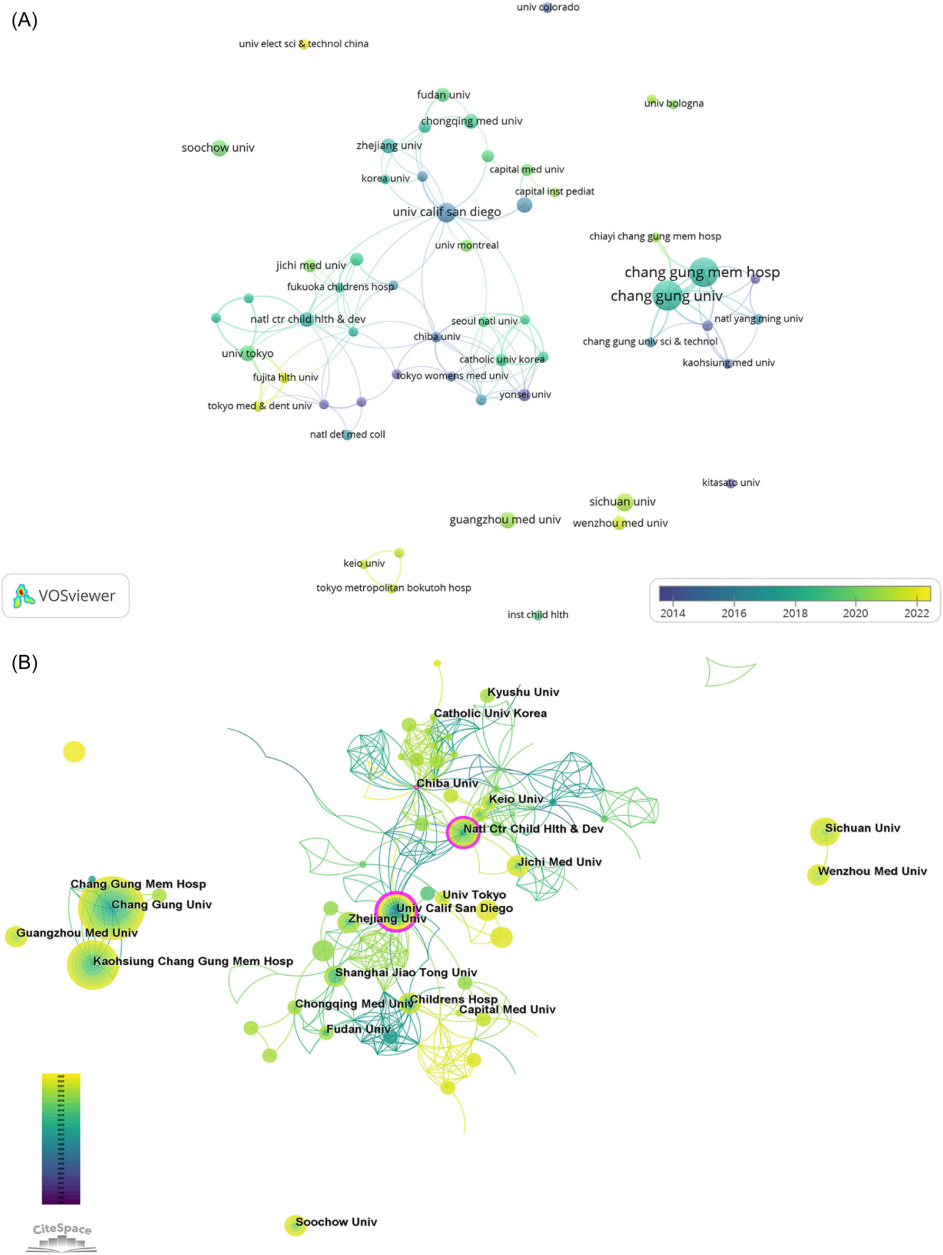
Analysis of institutions. (A) Analysis of cooperation networks between institutions. (B) Visual analysis of institutional collaboration in CiteSpace.

In VOSviewer, a total of 56 institutions had four or more articles on which we mapped the institutional collaboration network. According to total link strength, the top five institutions were Chang Gung University, Chang Gung Memorial Hospital, the University of Tokyo, the University of California, San Diego, and the National Center for Child Health and Development. There was a strong collaboration between Chang Gung University, Chang Gung Memorial Hospital, and Taipei Medical University, all of which are located in Taiwan (Figure 4A). A bias toward yellow indicates that the institution had been paying more attention to research in this field in recent years and was an emerging institution in this field. For example, Yonsei University (purple) and Tokyo Women's Medical University (purple) started research earlier, whereas Wenzhou Medical University (yellow) participated later in research in this field. In CiteSpace, Chang Gung University had the largest number of publications, but its centrality was low. On the contrary, the University of California, San Diego (0.25) and the National Center for Child Health and Development (0.18) had a higher centrality compared with other institutions, indicating that their research in the field occupied an important position (Figure 4B).

### 3.4 Analysis of journals

Articles related to IVIG nonresponse in KD were published in 143 journals, with *Frontiers in Pediatrics* (*n* = 33) being the most active journal in this field, followed by *Pediatric Rheumatology* (*n* = 19), *Journal of Pediatrics* (*n* = 16), and *Pediatric Infectious Disease Journal* (*n* = 15). *Journal of Pediatrics* (*n* = 1176) had the highest number of citations, with an average of 73.50 citations per article. The quality of journals can be assessed by analyzing the Journal Citation Reports (JCR) quartile, and Table 3 shows that 40% of the top 10 publications were in JCR quartile 1 (Q1) journals, 40% in Q2, and 20% in Q3.

**Table 3:** Top 10 journals in terms of number of publications.

Rank	Journal	Publications (*n*)	Citations (*n*)	Average number of article citations	Impact factor	JCR quartile
1	*Frontiers in Pediatrics*	33	205	6.21	2.6	Q2
2	*Pediatric Rheumatology*	19	211	11.11	2.5	Q2
3	*Journal of Pediatrics*	16	1176	73.5	5.1	Q1
4	*Pediatric Infectious Disease Journal*	15	325	21.67	3.6	Q1
5	*Plos One*	13	320	24.62	3.7	Q2
6	*European Journal of Pediatrics*	12	188	15.67	3.6	Q1
7	*Pediatric Cardiology*	12	107	8.92	1.6	Q3
8	*BMC Pediatrics*	11	122	11.09	2.4	Q2
9	*Clinical Rheumatology*	11	68	6.18	3.4	Q3
10	*Pediatric Research*	10	111	11.1	3.6	Q1

Abbreviation: JCR, journal citation reports.

**Figure 5:**
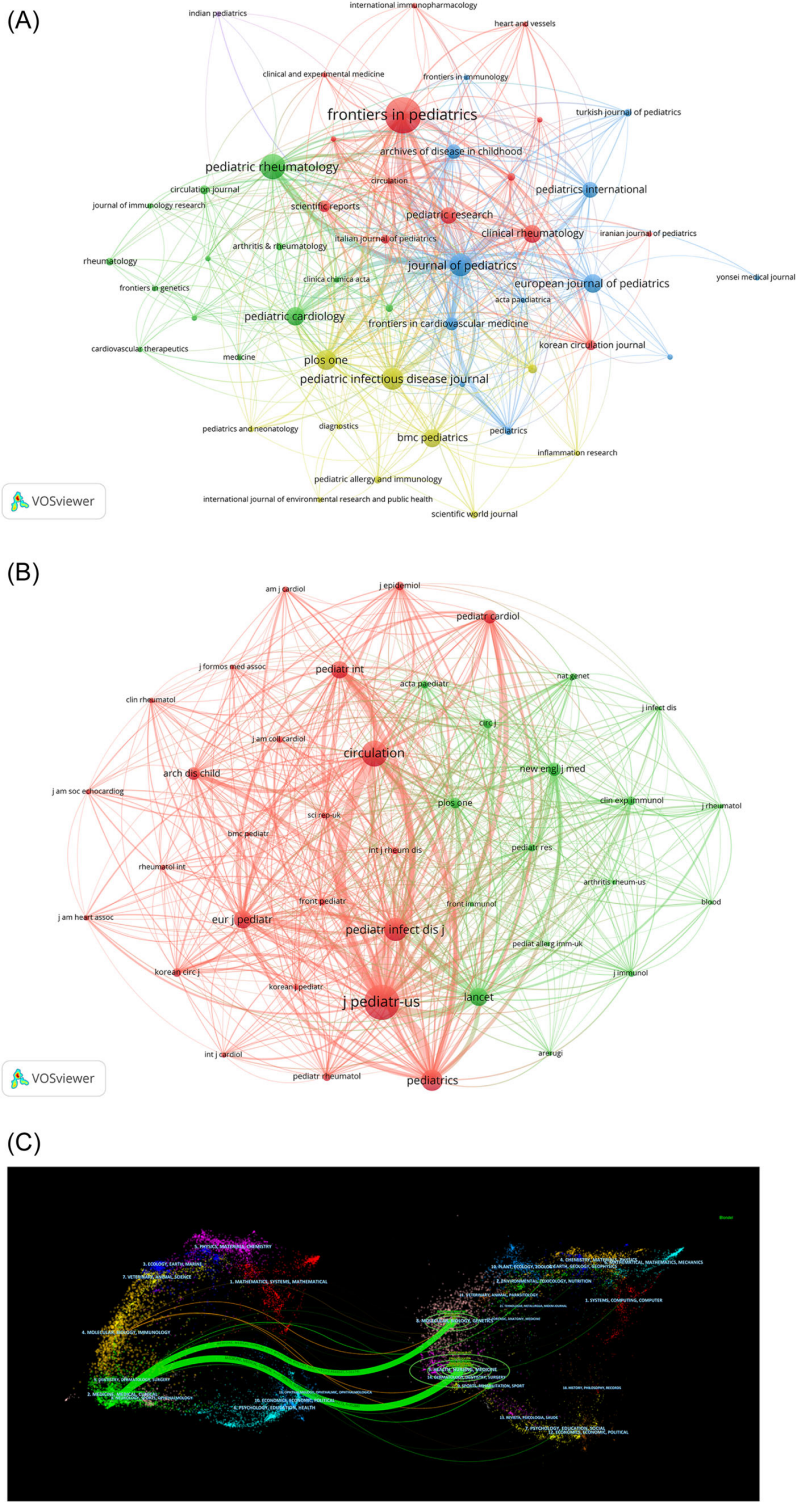
Analysis of journals. (A) Visual analysis of journals. (B) Visual analysis of co‐cited journals. (C) Dual‐map overlay of journals.

Subsequently, we filtered the journals with a minimum number of publications of two using VOSviewer and mapped the journal network (Figure 5A). *Journal of Pediatrics* had an active citation relationship with *Frontiers in Pediatrics, Pediatric Rheumatology, Clinical Rheumatology*, and *Pediatric Infectious Disease Journal*. The purpose of co‐citation analysis of journals is to capture the highly cited articles in the field and the journals that publish them. We analyzed the journals' co‐citation relationships using VOSviewer and set the threshold of the minimum number of co‐citations to 50. Figure 5B shows the co‐cited journals divided into two clusters, and the top five journals in terms of co‐citation frequency were the *Journal of Pediatrics* (*n* = 1164), *Circulation* (*n* = 746), *Journal of Pediatric Infectious Disease* (*n* = 631), *Journal of Pediatrics* (*n* = 544), and *Lancet* (*n* = 418). In addition, the *Journal of Pediatrics* has a positive co‐citation relationship with *Circulation, European Journal of Pediatrics, Pediatric Infectious Disease Journal, Lancet*, and *Pediatric*. In the dual‐map overlay of journals (Figure 5C), articles published in medicine/medical/clinical journals mainly cited articles published in journals of molecular/biology/genetics and health/nursing/medicine.

### 3.5 Analysis of authors

In VOSviewer, we conducted an analysis of the number of published articles and co‐citation frequency of authors (Table 4) to visualize the research strength of authors. A total of 1889 authors had contributed to the field of IVIG resistance in KD. Among the top 10 authors, Kuo Ho‐Chang had the most publications (*n* = 32), followed by Huang Ying‐Hsien (*n* = 17) and Lo Mao‐Hung (*n* = 12). Jane C. Burns had the highest number of citations (*n* = 660). The most frequently co‐cited authors were Jane W. Newburger, followed by Kobayashi Tohru and Jane C. Burns. Notably, Jane C. Ho‐Chang and Jane C. Burns ranked in the top 10 for both the number of publications and frequency of co‐citations, demonstrating their significant influence in the field.

**Table 4:** The top 10 authors in terms of number of publications and co‐citation frequency.

Rank	Author	Publications (*n*)	Citations (*n*)	Rank	Co‐cited author	Co‐citations (*n*)
1	Kuo, Ho‐Chang	32	640	1	Newburger, Jane W.	425
2	Huang, Ying‐Hsien	17	226	2	Kobayashi, Tohru	403
3	Lo, Mao‐Hung	12	175	3	Burns, Jane C.	314
4	Burns, Jane C.	11	660	4	Kuo, Ho‐Chang	231
5	Chang, Ling‐Sai	11	98	5	Tremoulet, Adriana H.	229
6	Hua, Yimin	11	76	6	McCrindle, Brian W.	221
7	Liu, Lei	11	76	7	Egami, Kimiyasu	172
8	Tremoulet, Adriana H.	11	641	8	Sano, Tetsuya	144
9	Yu, Hong‐Ren	11	343	9	Sleeper, Lynn A.	115
10	Zhou, Kaiyu	11	76	10	Kawasaki, Tomisaku	113

**Figure 6:**
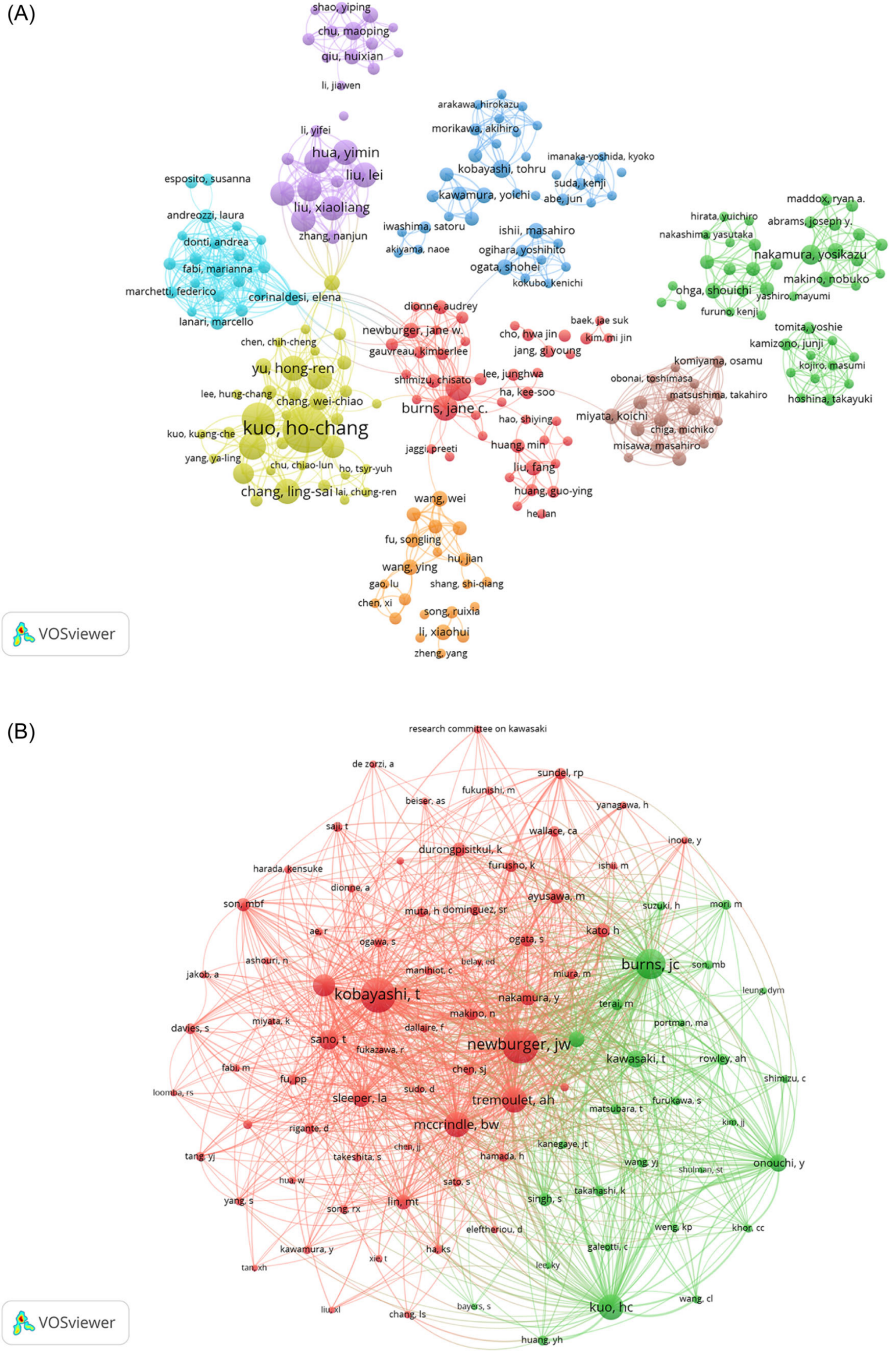
Analysis of authors. (A) Analysis of cooperation networks between authors. (B) Visual analysis of co‐cited authors.

We constructed an author collaboration network based on authors whose number of publications was two or more (Figure 6A). The 244 authors were categorized into eight clusters, indicating that there were eight main research teams. We found that researchers were more likely to cooperate within their own clusters. For example, the largest cluster, in red, is led by Jane C. Burns (United States), the yellow cluster is led by Kuo Ho‐Chang (Taiwan, China), and the purple cluster is Wang Chuan (Chinese Mainland). The result suggested that cooperation between clusters needs to be expanded and that there is a lack of close global collaboration. Similarly, among the 3982 co‐cited authors, we filtered the authors with co‐citations greater than or equal to 20 (Figure 6B). As shown in Figure 6B, this produced two clusters with the same color, indicating a close connection. Active collaboration between different co‐cited authors, such as Kobayashi Tohru, Jane C. Burns, and Jane W. Newburger, was observed.

### 3.6 Analysis of references

**Figure 7:**
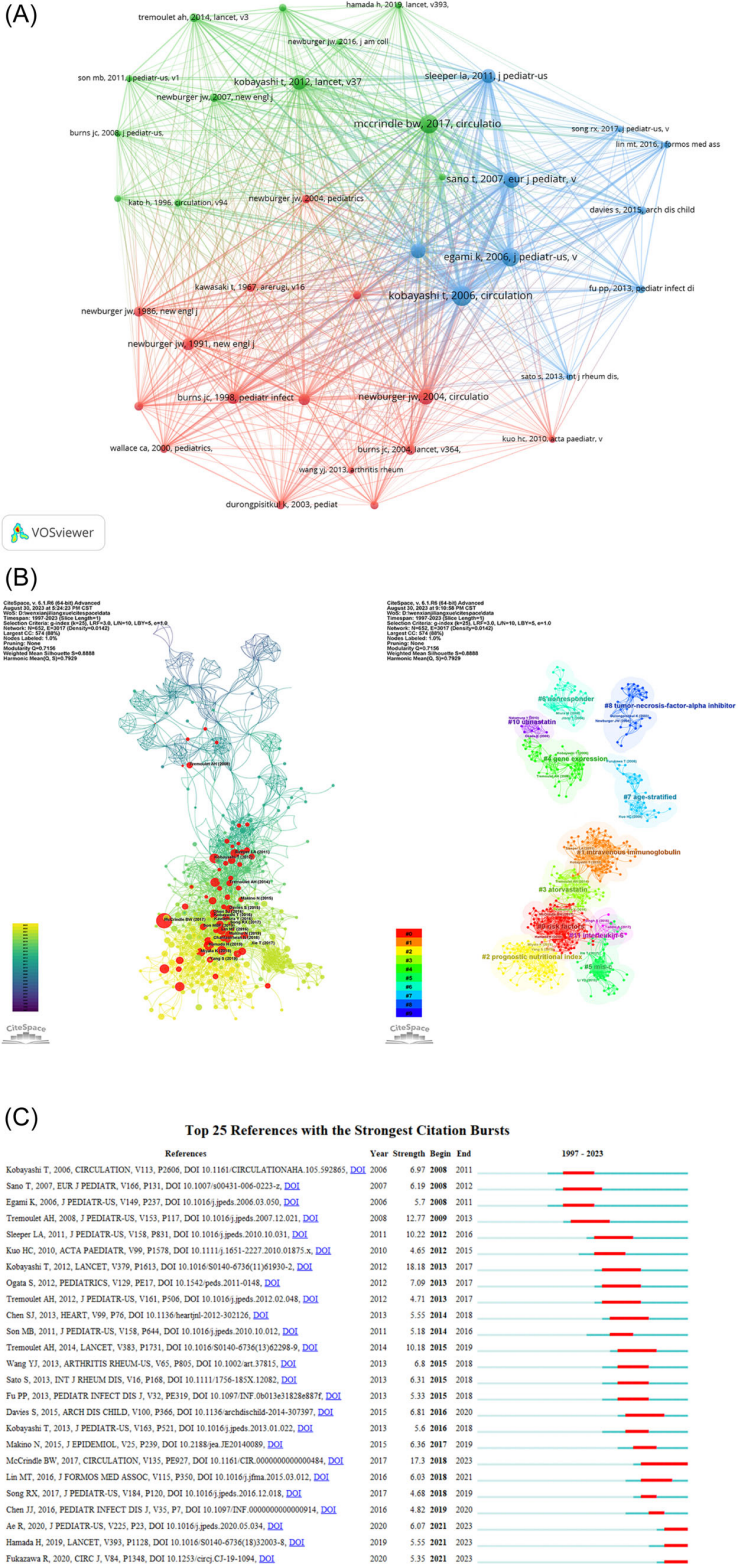
Analysis of references. (A) A collaborative network of co‐cited references. (B) Cluster analysis of co‐cited references. (C) The top 25 references with the strongest citation bursts.

Two articles cited by a third article at the same time are referred to as co‐citation of articles, and this can be used to analyze the relationships between the articles. We found 5159 co‐cited references for IVIG insensitivity in KD. We selected references with at least 30 co‐citations and constructed a co‐citation network using VOSviewer (Figure 7A). As shown, there was a strong co‐citation relationship between “kobayashi t, 2006, circulation,”^20^ “egami k, 2006, j pediatr us,”^21^ and “sano t, 2007, eur j pediatr.”^22^ Egami, Kobayashi, and Sano scoring systems are used to predict IVIG resistance and are the basis of research in this field. However, due to differences in race and population regions, the effectiveness of these scoring models still needs to be verified.^23^

At the same time, the references were clustered in CiteSpace according to similarity between them and divided into 42 clusters according to the log‐likelihood rate algorithm. The *Q*‐value was 0.7156 and *S*‐value 0.8888, indicating that the clustering results were reliable. As shown in Figure 7B, the largest 10 clusters were “risk factors,” “intravenous immunoglobulin,” “prognostic nutritional index,” “atorvastatin,” “gene expression,” “mis‐c,” “nonresponder,” “age‐stratified,” and “tumor necrosis factor‐alpha inhibitor.”

Figure 7C shows the top 25 references with the strongest citation bursts. Citation burst refers to the most frequently cited literature in a period of time, which can reflect the references that scholars are interested in. The article with the strongest citation burst (strength = 18.18) was “Efficacy of immunoglobulin plus prednisolone for prevention of coronary artery abnormalities in severe Kawasaki disease (RAISE study): A randomized, open‐label, blinded‐endpoints trial.” This article points out that early use of prednisolone can reduce the incidence of coronary artery abnormalities, shorten the duration of fever, and reduce inflammatory markers faster in patients predicted to be IVIG‐resistant.^24^ It is worth noting that the article published by McCrindle BW et al. in 2017 in Circulation, “Diagnosis, treatment, and long‐term management of Kawasaki disease: A scientific statement for health professionals from the American Heart Association,” also had a strong citation burst (strength = 17.3). The guidelines promote the diagnosis of incomplete KD, further improve the initial treatment of children who do not respond to IVIG and are at high risk of coronary artery abnormalities, and standardize the long‐term management of KD.^1^ The highest number of citation bursts occurred in 2013 (*n* = 5), indicating that research on IVIG resistance in KD was prominent that year.

### 3.7 Analysis of keywords

**Figure 8:**
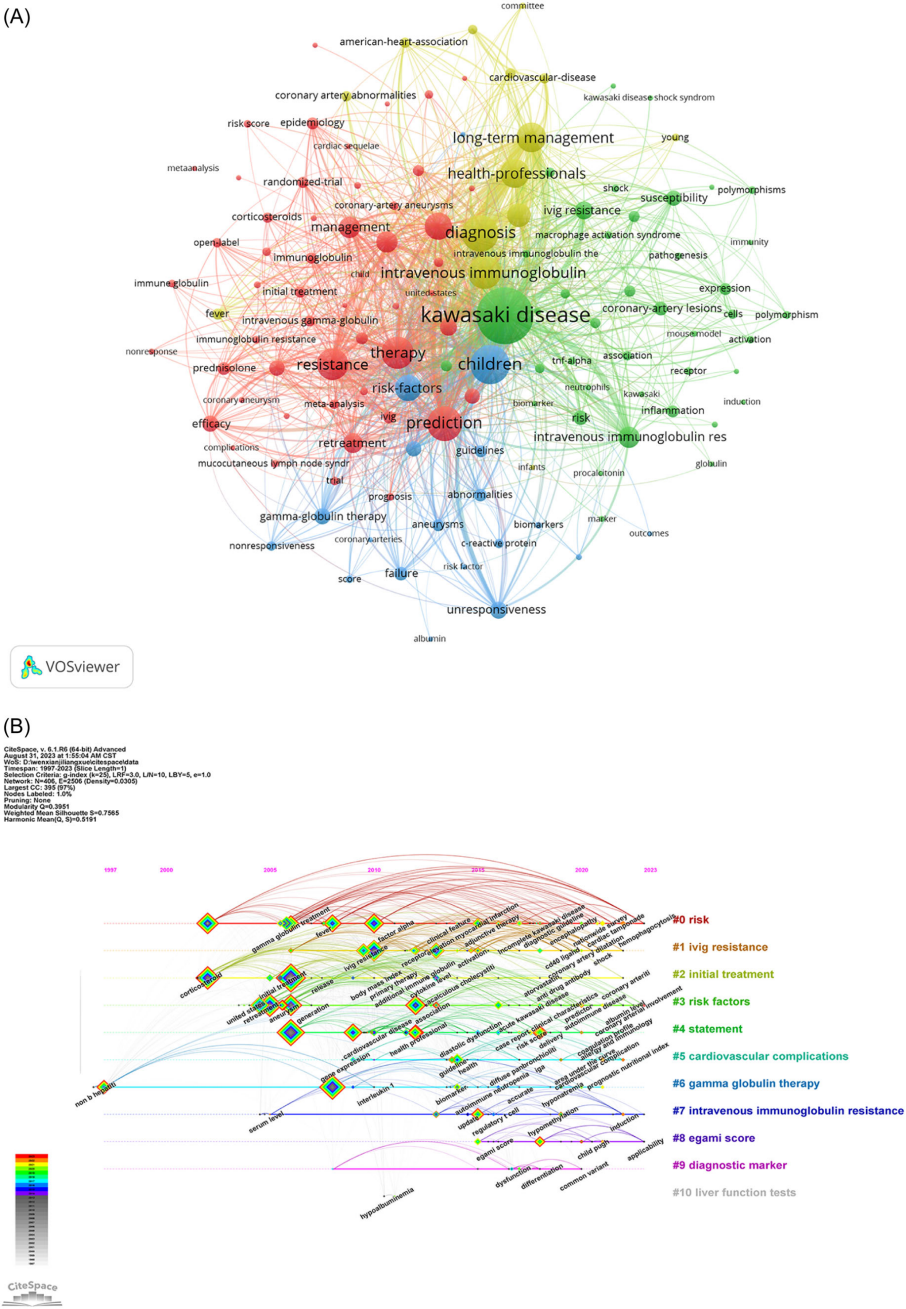
Analysis of keywords. (A) Collaborative network analysis of keywords. (B) Timeline view of keywords.

The analysis of keywords can provide the research hotspots in this field. Table 5 indicates the 20 keywords with the highest frequency and strongest centrality. Among them, the most frequently occurring words are KD (*n* = 225), children (*n* = 112), and IVIG (*n* = 96), which are all related to the subject terms of our search. In addition, the frequent occurrence of diagnosis (*n* = 107), prediction (*n* = 93), treatment (*n* = 84), and risk factors (*n* = 76) indicated that scholars in this field are mainly concerned with the diagnosis, treatment, model prediction, and risk factor screening of IVIG resistance in KD. The keyword with the highest centrality was gamma globulin (0.28), followed by gamma globulin therapy (0.12), coronary artery aneurysm (0.12), children (0.11), management (0.1), and coronary artery lesion (0.1). We screened keywords with a frequency greater than or equal to 5, obtained 126 keywords, and constructed a keyword collaboration network using VOSviewer. As shown in Figure 8A, the larger the node, the more frequently the keyword appeared, and the thicker the line, the stronger the connection between the keywords. Finally, the keywords were divided into four clusters, representing different research directions. The keywords in the red cluster focus on treatment (IVIG, prednisolone, anakinra, infliximab) and cardiovascular abnormalities. The keywords in the green cluster focus on mechanisms/pathophysiology and laboratory markers. The blue cluster focuses on risk factors. Yellow clusters are related to diagnosis and long‐term management.

**Table 5:** The top 20 keywords for frequency and centrality.

Rank	Keyword	Count (*n*)	Rank	Keywords	Centrality
1	Kawasaki disease	225	1	Gamma globulin	0.28
2	Children	112	2	Gamma globulin therapy	0.12
3	Diagnosis	107	3	Coronary artery aneurysm	0.12
4	Intravenous immunoglobulin	96	4	Children	0.11
5	Prediction	93	5	Management	0.1
6	Therapy	84	6	Coronary artery lesion	0.1
7	Risk factor	76	7	Intravenous immunoglobulin	0.09
8	Resistance	76	8	Coronary artery abnormality	0.09
9	Long term management	76	9	Unresponsiveness	0.08
10	Health professional	75	10	Susceptibility	0.08
11	Gamma globulin	65	11	Prediction	0.08
12	Coronary artery abnormality	65	12	IVIG resistance	0.08
13	Coronary artery lesion	61	13	Intravenous immunoglobulin resistance	0.08
14	Statement	55	14	Efficacy	0.07
15	Intravenous immunoglobulin resistance	44	15	Risk factor	0.06
16	Management	43	16	Cell	0.06
17	Retreatment	39	17	Risk	0.05
18	Coronary artery aneurysm	32	18	Retreatment	0.05
19	IVIG resistance	31	19	Intravenous immunoglobulin treatment	0.05
20	Unresponsiveness	30	20	Genome‐wide association	0.05

The timeline view reflects the progress of topics in this field over time, and Figure 8B is a timeline graph of keywords related to IVIG resistance from 1997 to 2023. The earliest cluster is #6 (gamma globulin therapy), which is relevant to our research topic, and the largest cluster is #0 (risk). Clusters #0 (risk) and #3 (risk factors) focus on identifying high‐risk patients susceptible to IVIG resistance and risk factors for developing coronary artery lesions. Notably, #0 (risk), #3 (risk factor), #7 (IVIG resistance), and #8 (Egami score) are four clusters that are ongoing, providing reference for research hotspots. In addition, #8 (Egami score) is the most recent cluster: the Egami score system is the traditional IVIG resistance prediction model, which is suitable for the Japanese population but is not very beneficial for the non‐Japanese population. Therefore, in recent years, new machine learning (ML) methods and high‐throughput sequencing have been applied to the prediction of IVIG resistance, indicating that the prediction of IVIG resistance remains a hot research topic.^25^

## 4 DISCUSSION

### 4.1 General information

Overall, the number of publications on the topic has been increasing year by year. From 1997 to 2009, there were relatively few articles on IVIG resistance in KD, and research in this field was still in its early stages. From 2009 to 2018, the annual number of publications gradually increased, indicating that more scholars were paying attention to the importance of IVIG resistance research. From 2019 to 2022, the number of publications increased significantly, which may have been due to the COVID‐19 pandemic, when a large number of children had KD‐like symptoms.^26^, ^27^ What is more, we noticed a rapid increase in the number of publications and citations in 2019, during which a phase III clinical trial in Japan confirmed that initial treatment with IVIG and cyclosporine (CSA) improved the prognosis of coronary artery injury.^28^

Through visual analysis of countries/regions and institutions, it could be seen that China, Japan, and the United States were the main countries/regions studying IVIG resistance in KD, and the top 10 institutions in terms of publications were also located in these three countries. A large number of articles have been published in China, but large‐scale multicenter research and high‐quality original research from the country are lacking. Owing to the professional title evaluation system, researchers have neglected deep thinking and original research, blindly pursuing the quantity of articles. In addition, China's research and medical institutions are widely distributed, but medical resources are scattered, making large‐scale multicenter cohort studies difficult. China will need to promote exchanges and cooperation among institutions to enhance high‐quality research in the future. The United States has top research institutes and universities, and the US government has provided sufficient research funding. The development of two KD management guidelines has improved the diagnosis and adjuvant treatment of refractory KD,^1^, ^29^ which, together with the construction of the San Diego Score in 2008,^30^ has placed the United States at the center of IVIG resistance research. It will continue to play a leading role in future research. In Japan, a phase III trial of the effect of CSA combined with IVIG therapy on coronary outcomes in IVIG‐resistant children has been conducted in recent years.^28^ In addition, a large national epidemiologic survey was conducted in which 19.7% of patients were found to have not responded to initial IVIG.^31^ The Japanese Circulatory Society and the Japanese Society of Cardiac Surgery jointly published guidelines detailing the diagnosis and long‐term management of cardiovascular sequelae of KD, contributing significantly to the advancement of KD management.^32^ France ranked first in citations per article but had a low number of publications. If France wished to enhance its international influence, it would need to increase the number of publications in the field of KD IVIG resistance. As shown in Figure 3A,B, there was active cooperation between China, the United States, and Japan, countries that also have a high prevalence of KD. Cooperation between countries has jointly promoted research on the treatment of IVIG‐resistant KD patients. For example, a meta‐analysis conducted by American and Chinese scholars was published in *JAMA Pediatrics*, which showed that for patients who did not respond to IVIG, a combination of corticosteroids and IVIG was more effective in improving coronary artery prognosis than IVIG alone.

In terms of research institutions, most of them were limited to domestic cooperation with less international cooperation, which is not conducive to long‐term academic development. We found that Chang Gung University published the most papers (*n* = 32), but the institution with the highest total citations and citations per article was Boston Children's Hospital in the United States. The University of California, San Diego in the United States had the highest centrality (0.25), followed by the National Center for Child Health and Development in Japan (0.18), suggesting that these two institutions occupy an important position in international cooperation. However, we note that Sichuan University, Guangzhou Medical University, and Soochow University, which had a high number of publications, had not collaborated with other institutions, potentially hindering the development of study in this field. Therefore, we strongly recommend increased cooperation and communication among various institutions to promote the development of research related to IVIG resistance in KD. From the perspective of journals, *Frontiers in Pediatrics* published the most papers, with 33 published articles, followed by *Pediatric Rheumatology* with 19. The dual‐map overlay of the journals suggested that current research is mainly focused on clinical research, with less emphasis on basic research.

In terms of authors, Kuo Ho‐Chang was the most productive, with 32 publications, and his main contribution was to explore laboratory predictive indicators^33^, ^34^ and genetic susceptibility^35^, ^36^ to IVIG resistance. Meanwhile, Kuo Ho‐Chang was also ranked fourth in terms of co‐citation frequency, which indicates his outstanding influence in the field of IVIG resistance. In addition, the author's collaboration network map showed that Kuo Ho‐Chang, Mao‐Hung, and Ying‐Hsien, all of whom are scholars from the Chang Gung Memorial Hospital in Taiwan, have close cooperative relationships. The most frequently co‐cited author was Jane W. Newburger (*n* = 425), followed by Kobayashi Tohru (*n* = 403). Jane W. Newburger conducted a systematic review of KD^37^ and revised the American Heart Association (AHA) guidelines in 2017. The guidelines updated the diagnosis, treatment, and long‐term management of KD and summarized additional treatment options for IVIG‐resistant KD patients.^1^

### 4.2 Research status and hotspots

Valuable publications can have a significant academic impact on research in the field. The analysis of keywords allowed us to summarize the most frequently researched topics in this field. Using CiteSpace, we screened the top 20 keywords with the highest frequency and centrality. After removing keywords related to the search terms, we found that research in the field of IVIG resistance had focused on the following three aspects:

#### 4.2.1 Diagnosis and treatment of IVIG resistance

The combination of high‐dose IVIG and aspirin is the initial treatment for KD, but up to 20% of patients do not respond to IVIG therapy, presenting with persistent or recurrent fever 24−48 h after completion of the first dose of IVIG, and an increased risk of coronary artery abnormalities.^38^, ^39^ According to the guidelines developed by the American College of Rheumatology in 2021, glucocorticoids or other immunomodulatory immunosuppressants in combination with IVIG are recommended for the treatment of IVIG‐resistant KD.^40^ Several trials have been conducted to study the treatment of steroids in high‐risk IVIG‐resistant patients. Kobayashi et al. administered prednisolone intravenously in three divided doses (2 mg/kg), the drug was changed to being taken orally after the fever subsided, and then, when C‐reactive protein returned to normal (< 5mg/L), the prednisolone dosage was gradually reduced in steps of 5 days.^24^, ^41^ Ogata et al. used methylprednisolone pulse therapy (30 mg/kg, 2 h) to treat refractory KD.^42^ Although steroid adjuvant therapy for high‐risk IVIG resistance is different, a meta‐analysis by Chen et al. found that for patients with high‐risk IVIG resistance, IVIG plus steroid therapy could reduce the incidence of coronary artery abnormalities.^43^

To date, several reviews and meta‐analyses have summarized various treatment approaches for IVIG‐resistant children with KD. However, most meta‐analyses have focused on comparing the efficacy and adverse event rates of different treatments in clinical trials and have lacked comprehensive evaluation of the dynamic changes in treatment regimens in the field. In this study, the references with the strongest citation bursts suggest the history of the development of IVIG resistance therapy. Adjuvant therapy for IVIG resistance has gone through a progression from infliximab in 2011,^44^ to intravenous corticosteroids in 2012,^24^ followed by corticosteroid pulse therapy.^42^ In 2012, calcineurin inhibitors such as CSA were used in the treatment of refractory KD.^45^ In 2019, a randomized open‐label phase III trial showed that CSA combined with IVIG reduced the incidence of coronary artery abnormalities in patients with KD who did not respond to predicted IVIG.^28^

The reference clustering analysis in our study showed that researchers were also very interested in atorvastatin, which may become a future research hotspot. Atorvastatin has anti‐inflammatory and antioxidant effects and a protective effect on the maintenance of vascular endothelial homeostasis, which is potentially valuable in the treatment of vasculitis. A recent study has shown that in KD, atorvastatin reduces the production of inflammatory mediators in vascular endothelial cells and prevents the proliferation of myofibroblasts, which may help to stop the development of coronary artery aneurysms.^46^ In addition, in a mouse model of Kawasaki vasculitis, atorvastatin was shown to reduce TNF‐α release and decrease matrix metalloproteinase‐9 secretion.^47^ However, the use of atorvastatin in IVIG resistance patients with KD has not been clinically tested and requires further study. In addition, biologics such as the interleukin‐1 receptor blocker anakinra^48^ have been used in the treatment of IVIG‐resistant patients. Plasma exchange and methotrexate^49^ are used for additional treatment of such patients.

#### 4.2.2 High‐risk factors and predictive models for IVIG resistance

As shown in Figure 7A, the co‐cited references build the knowledge base of the field. Using VOSviewer, we constructed a collaborative graph of co‐cited references, and the top three most co‐cited references focused on predictive models of IVIG resistance in KD.^20^, ^21^, ^22^ These models have different sensitivities and specificities, and it is worth noting that these scoring systems cannot be applied to different populations.^38^ Adriana H. Tremoulet, who established the San Diego predictive scoring system for IVIG resistance, which has a sensitivity of 73.3% and a specificity of 61.9%, had three reference citation bursts.^30^ Accurate identification of IVIG resistance is a major challenge in the treatment of KD, and early identification of IVIG resistance is essential for timely additional treatment. Fever duration, white blood cell count, lymphocyte percentage, neutrophil/lymphocyte ratio, C‐reactive protein/albumin ratio, aspartate aminotransferase, hematocrit, albumin, total bilirubin, lactate dehydrogenase, creatinine, and sodium have been used to predict IVIG resistance in KD in several studies.^38^, ^39^, ^50^ Based on laboratory findings and clinical features at the time of initial treatment, three IVIG risk prediction models have been established by logistic regression analysis and verified externally.^20^, ^21^, ^22^ ML can handle nonlinear input variables and therefore sometimes outperforms traditional models.^51^, ^52^ Currently, ML is used in clinical diagnosis and outcome prediction in many medical fields.^53^, ^54^, ^55^ The diagnostic criteria for IVIG resistance in KD are based on clinical parameters, and while traditional predictive models can incorporate only a small number of clinical features, models computed by ML can integrate all aspects of clinical indicators, including continuous variables, without the need for categorization. Additionally, ML models can encompass a wider array of predictors, such as genetic expression, enhancing their predictive capacity. ML also allows for the model to be easily retrained and updated with the most recent data, facilitating adaptability, and ongoing refinement. ML seems to be an effective tool for predicting IVIG resistance in children with KD.^56^, ^57^ Sunaga et al. used ML to predict the IVIG resistance of 1002 KD cases and identified the three most important influencing factors. It is worth noting that this scoring system is as reliable as the three traditional IVIG resistance scoring systems.^58^ The application of high‐throughput sequencing technology has enabled the integration of clinical variables with genotype and has been used to predict IVIG resistance.^59^ However, a meta‐analysis of 161 prediction models from 48 studies found that the validity of the existing prediction models for external validation was not clear, and it was still necessary to further develop and validate a reliable IVIG resistance prediction model.^60^

#### 4.2.3 Impact of COVID‐19 on research

Children become less ill with COVID‐19 than adults but can still develop severe MIS‐C that requires hospitalization and can be life‐threatening.^61^ MIS‐C has clinical symptoms similar to those of KD, such as fever, conjunctivitis, rash, and multiple organ involvement.^62^ Epidemiologically, children with KD tend to be younger than 5 years of age and are concentrated in East Asian countries, whereas MIS‐C tends to occur in children older than 6 years of age, and mostly in Europe and the United States.^63^, ^64^ Bibliometrics shows that the United States, China, and Italy have dominated the studies of COVID‐19 in children.^63^, ^65^ In 2021, Henderson et al.^66^ published guidelines recommending IVIG and glucocorticoids as first‐line agents for patients with MIS‐C. IVIG‐insensitive MIS‐C is treated with bioimmunomodulators such as anakinra, tocilizumab, and siltuximab.^67^ Pediatric COVID‐19 research has focused on pathogenesis, public health, mental health, and treatment.^68^ Morand et al.^69^ demonstrated a dramatic increase in the number of publications related to Kawasaki‐like disease caused by the COVID‐19 epidemic; similarly, our bibliometric analysis found a rapid increase in publications related to IVIG resistance in 2020 and that MIS‐C was a hot research topic.

### 4.3 Future research frontiers

Understanding the pathogenesis of IVIG resistance in KD and the accurate identification of patients at risk remain critical challenges that will define future research directions. Genetic susceptibility has emerged as a pivotal area of investigation. The application of high‐throughput sequencing technologies has begun to unravel numerous genetic markers linked to IVIG resistance, such as inositol 1,4,5‐trisphosphate 3‐kinase C (ITPKC). These discoveries are crucial to elucidating the mechanisms underlying IVIG resistance and could facilitate the early identification of high‐risk patient groups.

Moreover, the rapid expansion of data volume in the digital era has necessitated the adoption of advanced computational techniques. ML, in particular, is well‐suited for handling the complex and voluminous clinical data typical of KD. While traditional scoring systems based on logistic regression have been utilized to model clinical and laboratory parameters of KD, their limited sensitivity often results in a significant number of children with IVIG resistance being overlooked. ML offers a substantial improvement in this area, providing the ability to incorporate and analyze large‐scale datasets to build reliable and generalizable predictive models.

Exploring potential targets for therapeutic intervention in IVIG resistance remains a hot topic. Ongoing research into IVIG resistance‐associated genomics, supported by advances in sequencing technologies, continues to identify viable genetic targets. Previous genome‐wide association studies have pinpointed relevant genes, paving the way for integrating genotypic variables with clinical indicators to enhance the accuracy of prediction models.

The integration of these genetic and computational approaches will likely revolutionize the prediction and management of IVIG resistance in KD. As such, our future research will focus on harnessing these innovative technologies to improve diagnostic and therapeutic strategies, thereby significantly impacting the field's global research landscape.

## 5 ADVANTAGES AND SHORTCOMINGS

This study has some advantages. First of all, a bibliometric analysis of the field of IVIG resistance in KD was conducted for the first time, providing a comprehensive summary for this field. Second, four tools were used: CiteSpace, VOSviewer, R package Bibliometrix, and Microsoft Office Excel 2019, and the analysis was objective and comprehensive. However, there were some limitations. First, only the content of the WoSCC database was searched and some articles from other databases were ignored, thus there was some selection bias. Second, we only searched for literature published in English and literature that was either articles or reviews.

It is important to note several factors that may influence our bibliometric analysis: (1) Variability in diagnostic criteria: Diagnostic standards for KD and IVIG resistance differ significantly across regions and institutions, which can affect research trends. For instance, while Japan uses criteria from its Ministry of Health, most other countries follow those set by the AHA. (2) Digital communication: The digital era has facilitated easier and more effective communication, enhancing collaboration across borders and institutions. (3) Economic factors: The costs associated with publishing, including author processing fees and open access policies, influence where and how frequently research is published. These costs also affect collaborative dynamics and the visibility of research through cooccurrence and co‐citation patterns.

## 6 CONCLUSIONS

A bibliometric analysis of 364 publications was performed to provide information on the study of IVIG in KD. More and more countries, institutions, and scholars are showing strong interest in this field. The hotspots and frontiers of research in this field are still the diagnosis and treatment of IVIG‐resistant patients, the identification of risk factors that predispose to IVIG resistance, and the construction of predictive models for IVIG resistance. We hope that this study will help scholars understand the comprehensive knowledge architecture of the field.

## AUTHOR CONTRIBUTIONS

**Jiaying Zhang and Hongbiao Huang**: Conceptualization. **Jiaqi Jiang**: Data curation. **Xuan Tang**: Formal analysis. **Yan Wang**: Investigation. **Lei Xu**: Methodology. **Shuhui Wang**: Software. **Haitao Lv**: Supervision. **Yang Gao, Wenyu Zhuo, and Yiming Zheng**: Validation. **Lei Xu and Shuhui Wang**: Visualization. **Jiaying Zhang**: Writing—original draft. **Hongbiao Huang**: Writing—review and editing.

## CONFLICT OF INTEREST STATEMENT

The authors declare no conflict of interest.

## Data Availability

Data may be consulted from the corresponding authors for reasonable reasons.
